# Feasibility of a Motor-Cognitive Stepping Training Program During Acute Hospitalization in Patients with Traumatic Brain Injury

**DOI:** 10.2147/TCRM.S599343

**Published:** 2026-09-30

**Authors:** Julia Brechbühl, Anne-Katrin Hickmann, Erik Schulz, Alexandra Steurer, Eleftheria Giannouli

**Affiliations:** 1 School of Health Sciences, Institute of Physiotherapy, Zurich University of Applied Sciences, ZHAW, Winterthur, Switzerland; 2 Department of Neurosurgery, Cantonal Hospital & Medical School of St. Gallen, St. Gallen, Switzerland; 3 Centre for Ergo- and Physical Therapy, Cantonal Hospital of St. Gallen, St. Gallen, Switzerland; 4 Department of Health Sciences and Technology, Rehabilitation Engineering Laboratory, ETH Zurich, Zurich, Switzerland

**Keywords:** step training, dual-task training, neurological diseases, rehabilitation, balance

## Abstract

**Background:**

Traumatic brain injury (TBI) survivors often experience mobility issues which are further worsened by cognitive deficits. Dual-task training has shown promising effects in people with TBI. This study aims to evaluate the feasibility of the novel training concept “StepIt”, which addresses motor and cognitive falls-related aspects during acute hospitalization in TBI patients.

**Methods:**

Persons admitted to the hospital with a mild to severe TBI received individual training sessions of approximately 15 minutes per session during their hospital stay according to the “StepIt” training concept. The main outcome was feasibility as measured by rates of recruitment, inclusion, adherence, compliance, attrition, and safety. Secondary outcomes were training performance and user evaluation.

**Results:**

Rates for recruitment, adherence, and compliance were 72.9%, 87.5%, and 93.8%, respectively. No intervention-related adverse events occurred during the study period. The inclusion rate was 7.4% and attrition rate was 42.9%. Overall, four of the six predefined feasibility criteria were met, indicating that modifications to the study design are required before proceeding to a larger-scale study.

**Conclusion:**

The findings support the feasibility of StepIt during acute hospitalization in people with TBI, although modifications to the study design are needed. Future studies should examine its feasibility in different settings, such as inpatient or outpatient rehabilitation.

## Introduction

Traumatic brain injuries (TBIs) refer to functional disorders of the brain with or without demonstrable injuries to the brain as a result of external force to the skull.^1^ The most common causes are traffic accidents and falls.^2^ TBIs represent a prevalent issue worldwide, affecting 50–60 million individuals annually.^3^ Mild TBIs account for 70–90% of these cases, the remaining are moderate or severe TBIs.^2^,^4^ Recurrent TBI is in turn associated with high morbidity and mortality.^5^

TBI survivors commonly face mobility challenges related to walking due to impairments in motor skills such as gait stability, static and dynamic balance and motor coordination.^6^,^7^ It has been shown that individuals with TBI walk more slowly due to decreased step and stride length.^8^,^9^ In addition, individuals with TBI often have persistent cognitive impairments, including difficulties with attention, memory, and executive functions.^10^,^11^ These cognitive limitations can again adversely affect motor functions such as gait^12^ and increase the risk of falls, potentially leading to further injuries.^13^,^14^ Therefore, rapid improvement of motor and cognitive functioning and thus prevention of further falls and injuries are essential.

Research indicates that cognitive functions, particularly attention and executive function, are closely associated with physical functioning, including gait, mobility, and balance.^15^,^16^ Furthermore, incorporating cognitive demands into motor training may support motor recovery, with motor-cognitive training showing beneficial effects on gait, balance, motor function, and activities of daily living in neurological populations.^17^,^18^ Dual-task training, involving the simultaneous execution of two distinct tasks, such as walking while counting backwards, has shown efficacy in enhancing functional outcomes compared to conventional motor-only training.^19^ Moreover, dual-task training has demonstrated effectiveness in enhancing gait performance and decreasing the risk of fall in various neurological disorders like Parkinson’s disease and stroke.^20–24^ Although the underlying pathophysiology of these neurological diseases and TBI is different, it is reasonable to assume that patients with TBI would also benefit from motor-cognitive dual-task training. However, research on the effects of such training in patients with TBI is limited. To our knowledge, only two studies to date have looked into the effects of motor-cognitive dual-task training in people with TBI on various gait parameters such as step length, speed, static and reactive balance, dual-task performance, and cognition.^25^,^26^

Rehabilitation should begin as early as possible following traumatic injury, with rehabilitation needs assessed at hospital admission and interventions tailored to the individual patient. Early rehabilitation includes interventions targeting physical functioning and mobility, while cognitive impairments should also be identified and addressed as part of multidisciplinary care.^27^ However, structured interventions simultaneously targeting motor and cognitive functions during acute hospitalization after TBI remain scarce. This highlights the potential value of motor-cognitive training approaches that can be integrated into acute inpatient care.

In response to the demand for research on interventions tailored for individual recovery from TBI, which cover motor and cognitive abilities simultaneously, the recently developed training concept “StepIt” could be a relevant approach.^28^ “StepIt” is a step-based dual-task balance training concept designed to target both motor and cognitive aspects related to falls in which participants are presented with step patterns which they must memorize and execute to the pace of a metronome. The aim of this study is to assess the feasibility, training performance and user evaluation of “StepIt” during the acute hospitalization in patients with TBI.

## Materials and Methods

### Design

This study was a single-arm, monocentric intervention trial conducted at the Cantonal Hospital St. Gallen (Switzerland). The study design was approved by the Cantonal Ethics Committee Eastern Switzerland (ID: 2023–01472), it was registered at clinicaltrials.gov (ID: NCT06149975; Registration Date: 22-10-2023). The study was conducted according to the guidelines of the Declaration of Helsinki and reported in accordance with the CONSORT extension for randomized pilot and feasibility trials.^29^

### Participants

Recruitment was done upon admission at the Cantonal Hospital St. Gallen. Participants were screened by the local principal investigator according to the following criteria. Inclusion criteria were: age ≥18 years, diagnosis of mild to severe TBI according to the classification by the Glasgow Coma Scale,^30^ the physical ability to stand (using cane if needed), a minimum stay of >24h on regular ward, and the ability to give informed consent. Exclusion criteria were: limited vision and hearing ability (except facial injuries with monocular swelling), contraindications or inability to undergo the investigated intervention or score <16 on the Montreal Cognitive Assessment (MoCA). Eligible participants were finally included after providing written informed consent. A pragmatic target sample size of 12 participants was defined based on the number of patients with TBI admitted to the study site in previous years and the number of potentially eligible participants expected during the predefined five-month recruitment period. Recruitment would be stopped when the target size was reached, or the planned time frame expired.

### Intervention

The training sessions were delivered by physiotherapists already working in the hospital with a four-year BSc degree in physiotherapy and at least one year of professional experience in acute neurological care. Prior to study initiation, all participating physiotherapists received a one-hour standardized training session from the PI covering the StepIt intervention protocol and the relevant standard operating procedures and work instructions to ensure standardized intervention delivery. Participants received at least 3 to max. 10 individual and supervised training sessions of approximately 15 minutes per session during their stay at the hospital. A 90 × 90cm mat made from extra non-slip yoga mats was used. The mat was divided into nine equal squares with a 3 × 3 pattern (see Figure 1). Participants were presented with stepping patterns which they had to memorize and then execute on the mat in the pace given by a metronome. The session started with the therapist explaining and if needed demonstrating the pattern to the patient. After a couple of rounds practicing the pattern in their self-selected speed, a metronome was switched on and the patterns were executed by the participant to the given pace. All participants started at level one of totally three. Level one includes steps forward, sideways, and backward. At level two, steps across the midline are introduced, and at level three, additional crossover steps are added. The progression could be further adjusted individually through modification of the pattern’s length, the pace, and the execution with one or both legs. The switch to a new pattern occurs after approximately four correct completions of the current pattern. If the pattern is incorrectly executed multiple times, the therapist may decide to change the pattern, or it can be changed based on the patient’s request. The patterns manual can be seen in Supplementary Material 1.

**Figure 1:**
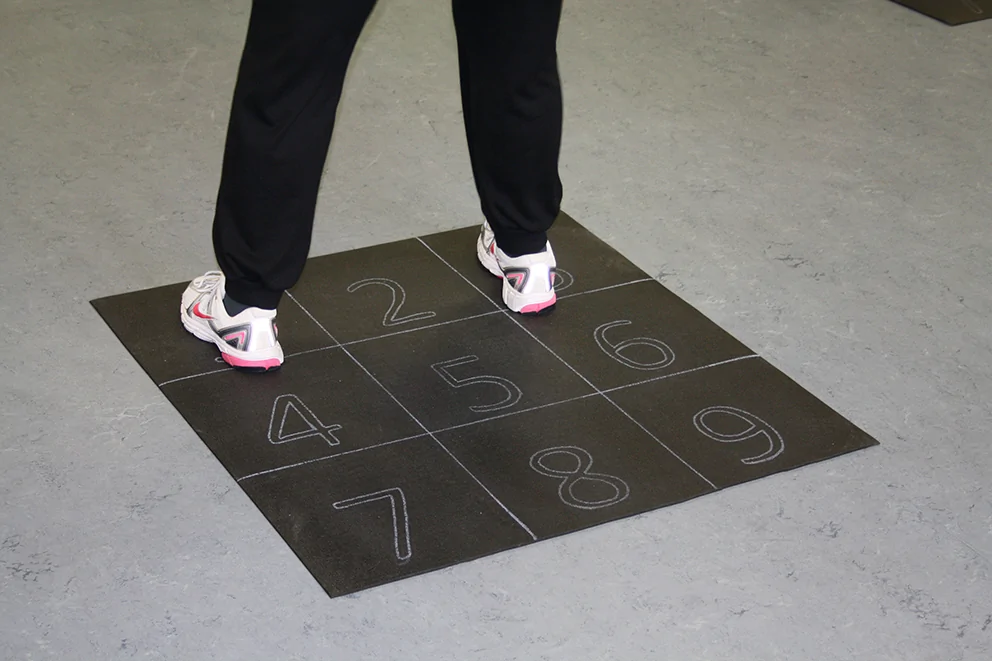
“StepIt” mat.

### Outcome Measures

The primary outcome of this study was feasibility defined as an umbrella term for the following measures: recruitment rate, inclusion rate, attrition rate, adherence rate, compliance rate, and safety. The recruitment rate was calculated by dividing the number of eligible participants by the total number of patients admitted with TBI during the study period. Inclusion rate was calculated by dividing the total number of included participants by the total number of eligible participants and the attrition rate by dividing the number of dropouts by the total number of included participants. Adherence and compliance rates were determined based on the total number of attended training sessions relative to the total number of offered training sessions, and the total attended training minutes relative to the total offered training minutes, respectively. Safety was assessed by counting the total number of (serious) adverse events. For feasibility, a protocol including criteria with thresholds was pre-defined (see Table 1), based on a comparable feasibility study.^31^ The secondary outcomes included user evaluation and training performance. User evaluation regarding physical and cognitive load was assessed using two questions of the NASA Task Load Index questionnaire after each training session.^32^ Cognitive and physical load were rated from 0 to 20, where 0 indicated “very low” and 20 “very high”. Patient satisfaction was measured using a tailored intervention-specific questionnaire completed after the final training session. The questionnaire includes questions with five possible answers each, ranging from “strongly agree” to “strongly disagree” and from “very low” to “very high”. Training performance, including improvement (Level 1–3 for step directions, length of correct patterns, beats per minute) and capacity (number of correct and incorrect patterns), was evaluated during each training session.

**Table 1:** Feasibility Criteria and Protocol

Feasibility Criteria	Definitions	Thresholds for Feasibility
Recruitment rate	= eligible participants/total patients with TBI	≥ 15%
Inclusion rate	= included participants/eligible participants	≥ 50%
Adherence rate	= attended/offered training sessions	≥ 80%
Compliance rate	= attended/offered training time	≥ 80%
Attrition rate	= dropouts/enrolled participants	≤ 10%
Safety	= number of (serious) adverse events	= 0
**Possible Scenarios:**	**Consequences**	
All feasibility criteria met	Intervention is feasible; subsequent study can be conducted without modifications	
Recruitment rate too low	Evaluate the reasons for failed recruitments: - reconsider recruitment procedure for subsequent study	
Inclusion rate too low	Evaluate the reasons for failed inclusion: - reconsider inclusion procedure for subsequent study	
Adherence rate too low	Evaluate the reasons for non-adherence - implement minor modifications for subsequent study	
Compliance rate too low	Evaluate the reasons for non-compliance - implement minor modifications for subsequent study	
Attrition rate too high	Evaluate reasons for dropouts: - in case of training-unrelated dropouts only, continue with subsequent study without modifications - in case of training-related dropouts, modifications must be undertaken to hinder similar events in the subsequent study	
Safety	In case of a training-related (serious) adverse event, the subsequent study must not be conducted	
≥ 2/6 feasibility criteria not met	Evaluate reasons and implement major modifications for the subsequent study	
≥ 5/6 feasibility criteria not met	Subsequent study must not be conducted	

### Statistical Analysis

All outcomes were analyzed calculating the rates, ranges and mean of values. In addition, a qualitative report was created out of the notes of the self-developed questionnaire. The statistical analysis was performed using the R/R-Studio version 2023.12.0+369 and Microsoft Excel version 16.83. Missing data were not replaced and data from withdrawn participants who terminated the intervention prematurely or had to be excluded during the study were used for analysis as far as possible since attrition rate was part of the feasibility. Dropout reasons were recorded if available.

The feasibility thresholds were set at ≥15% recruitment rate, ≥50% inclusion rate, ≥80% adherence rate, ≥80% compliance rate, ≤10% attrition rate and zero training related adverse events. The feasibility protocol (see Table 1) provided guidance on interpreting the results under various scenarios based on different feasibility outcomes as well as on how to proceed with the “StepIt” approach in a subsequent study.

## Results

### Participants Overview

Between November 2023 and March 2024, one hundred and twenty-nine patients with TBI have undergone eligibility screening. One hundred fourteen suffered a mild, nine a moderate and six a severe TBI. The most common reasons for exclusion were limited mobility (N = 13) and delirium or disorientation (N = 12). Seven out of ninety-four who fulfilled inclusion criteria gave written, fully informed consent for this study. One included participant had a moderate and six a mild TBI. Main reason for non-participation of patients who met the inclusion criteria was early hospital discharge after less than three days (N = 78). Of the initially included seven participants, four completed the required minimum of three trainings sessions while three dropped out of the study earlier. The reasons for dropout were that two participants were discharged after completing one and two training sessions respectively. The third participant no longer met the MoCA score required for inclusion. All three dropouts were diagnosed with a mild TBI. The flowchart of inclusion is shown in Figure 2 and sample characteristics are presented in Table 2.

**Table 2:** Sample Characteristics

Participant-ID	1	2	3	4	5	6	7	Mean
Age [years]	66	70	74	50	84	74	88	72.3
Weight [kg]	54	70	55	82	64	51	67	63.3
Height [cm]	158	158	155	175	154	157	168	160.7
Days since trauma	6	1	4	4	4	1	5	3.6
Severity	Moderate	Mild	Mild	Mild	Mild	Mild	Mild	-

**Figure 2:**
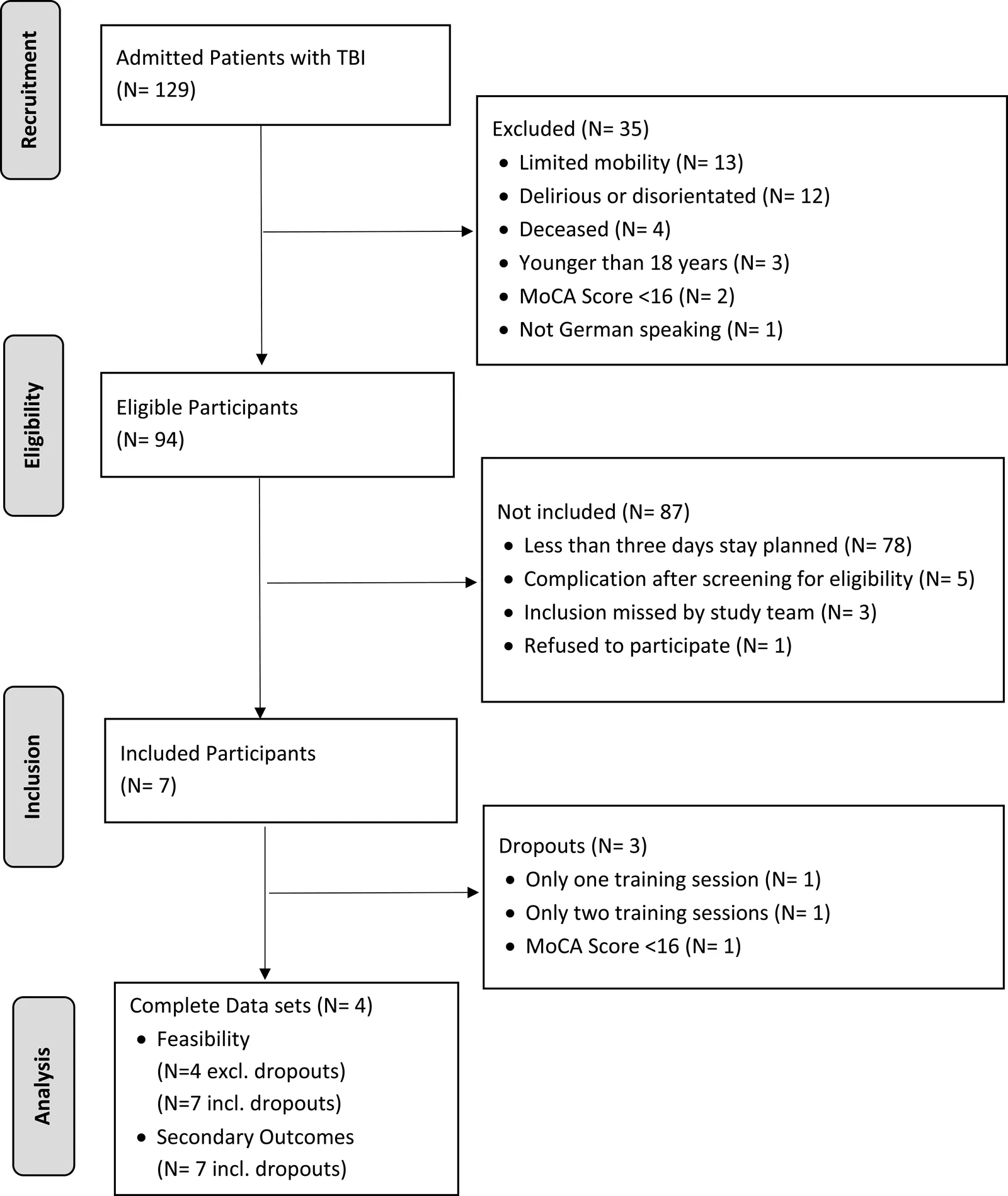
CONSORT diagram of the participant flow.

### Feasibility

Rates for recruitment, adherence, and compliance were 72.9%, 87.5%, and 93.8%, respectively. Taking the data from dropouts into account, the rates of adherence and compliance were 90% and 95.2% respectively. Over the study period no intervention-related serious adverse event occurred. In one participant, the occurrence of dizziness was recorded as an adverse event in two out of three training sessions. However, the rates for inclusion (7.4%) and attrition (42.9%) did not fulfill the pre-defined thresholds (see Table 3).

**Table 3:** Feasibility Outcomes

Feasibility Criteria	Data		Results	Threshold	Criteria Met
Recruitment rate	Admitted participants [number] eligible participants [number]	129 94	72.9%	≥ 15%	Yes
Inclusion rate	Eligible participants [number] included participants [number]	94 7	7.4%	≥ 50%	No
Adherence rate	Total offered trainings [number] total attended trainings [number]	16 14	87.5%	≥ 80%	Yes
Compliance rate	Total offered training time [min]* total attended training time [min]	210 197	93.8%	≥ 80%	Yes
Adherence rate**	Total offered trainings [number] total attended trainings [number]	20 18	90%	≥ 80%	Yes
Compliance rate**	Total offered training time [min]* total attended training time [min]	270 257	95.2%	≥ 80%	Yes
Attrition rate	Included participants [number] drop-outs [number]	7 3	42.9%	≤ 10%	no
Safety	Intervention related Serious Adverse Events [number]	0	0	none	Yes

**Notes**: *Not attended training sessions were not included. **Dropouts included.

### User Evaluation and Training Performance

#### User Evaluation

User evaluation regarding physical and cognitive load was assessed from fifteen out of eighteen attended training sessions. Participants reported a mean cognitive demand of 8.67 (SD = 4.88) and a mean physical demand of 8.67 (SD = 4.67), each on a scale ranging from 0 to 20. Excluding the user evaluation of the dropouts, data from eleven trainings sessions out of fourteen were available and the experienced physical load (mean score 8.64, ±4.78) was slightly higher than the cognitive load (mean score 7.73, ±5.04). The changes in cognitive and physical load within the participants across the training sessions are shown in Figures 3 and 4. The graphs include data from participants who completed at least two training sessions and for whom data on cognitive and physical load was available.

**Figure 3:**
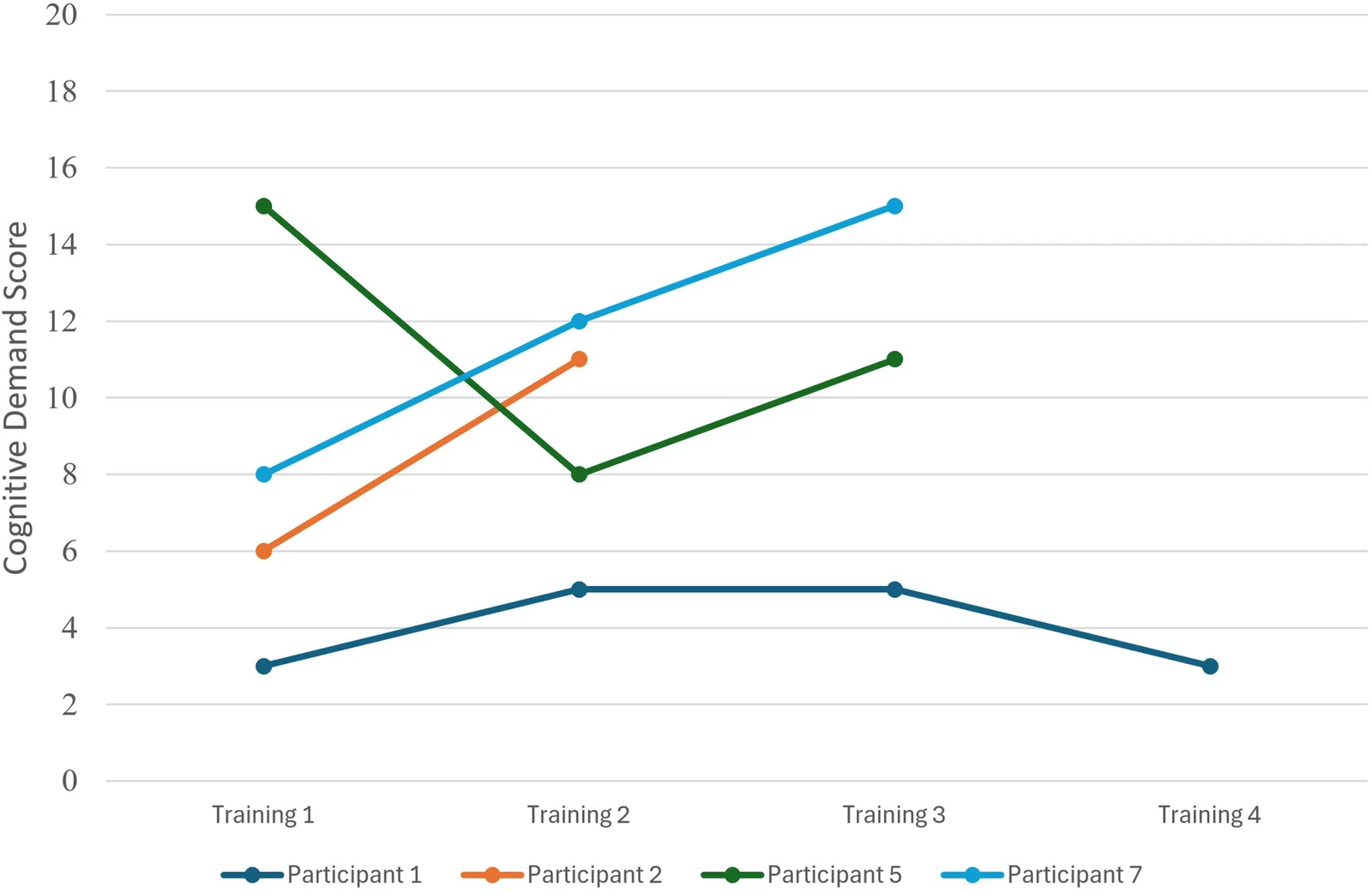
Changes in cognitive load across the training sessions for each participant.

**Figure 4:**
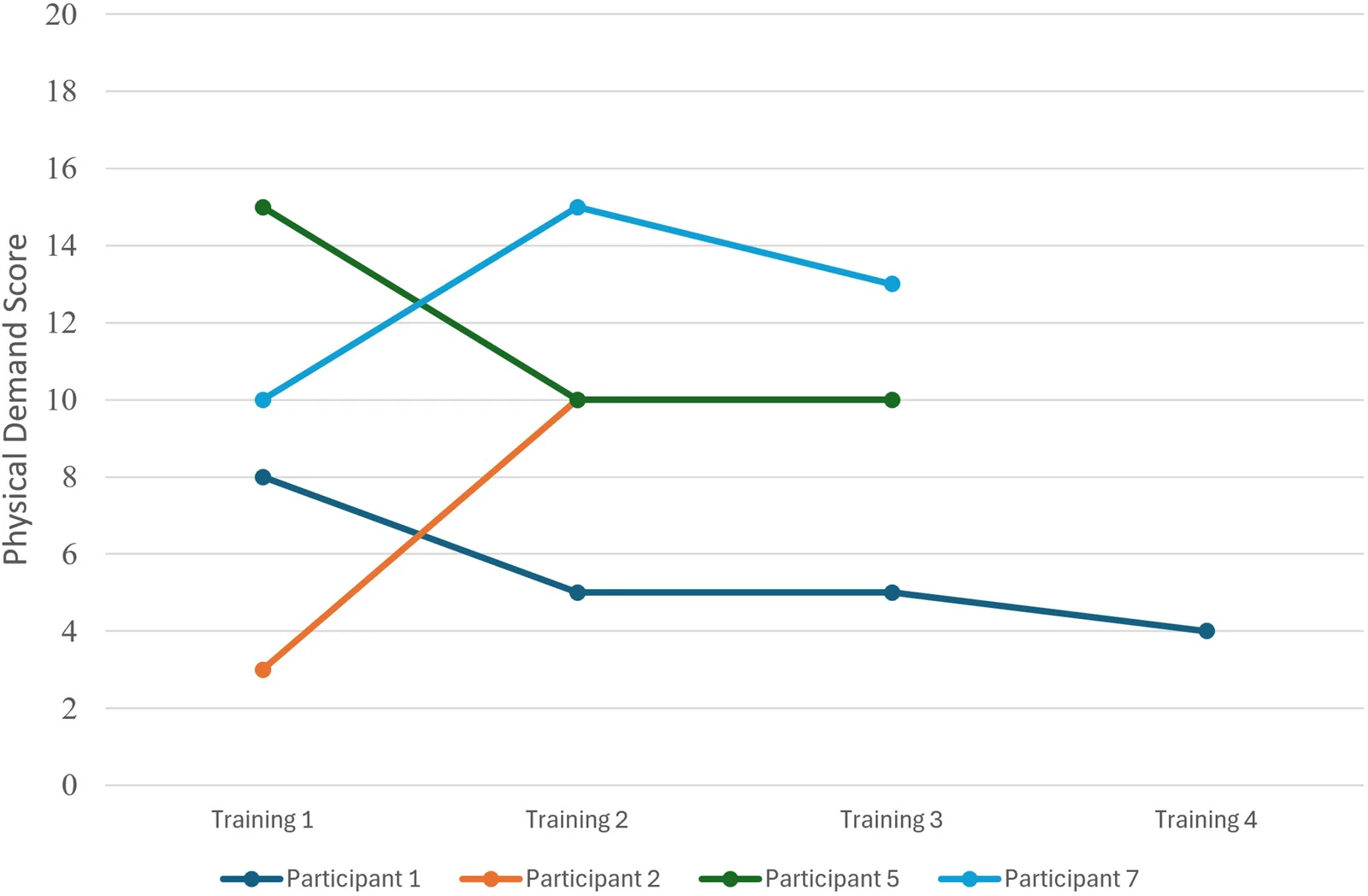
Changes in physical load across the training sessions for each participant.

User evaluation regarding patient satisfaction was recorded from four participants. Of the four participants who completed three full training sessions, three answered the questions on patient satisfaction. The fourth was unable to answer the questions due to insufficient language skills. The fourth questionnaire included in the analysis was from a participant who had dropped out after completing only two training sessions. The results show that all four participants found the training quite or very enjoyable. Additionally, three out of the four participants reported moderate improvements in coordination and balance. One participant rated the training as unfavorable due to experiencing increased dizziness during the first two out of three training sessions. Two participants noted a positive effect on cognition after the training with one of them also experiencing an increase in self-confidence. Except for one, all participants would recommend the training to others moderately to very highly. The intensity, frequency and duration of the training were also considered appropriate by the majority. The questions and their corresponding responses with their frequencies are shown in Table 4.

**Table 4:** Results of the Tailored Patient Satisfaction Questionnaire

Questions	Strongly Agree/Very Low	Agree/Low	Moderately Agree/Exactly Right	Disagree/High	Strongly Disagree/Very High
**I enjoyed** the training	1	2	1		
**The training improved coordination and balance**			3	1	
**The training was good for me**	2	1			1
**I noticed positive effects after the training**	2				2
**I would recommend the training to people with coordination or balance. Problems**	3				1
**I would recommend the training to people with memory problems**	2	1	1		
**I would generally recommend the training to others**	2	2			
**I would be interested in continuing training**	1	1			2
**How would you rate the intensity of the training?**			3	1	
**How would you rate the frequency of the training?**			3	1	
**How would you rate the duration of the training?**			4		

**Notes**: The values represent frequencies (absolute number of participants answering this question).

The therapists’ satisfaction with the training and its implementation was assessed using a tailored questionnaire. The results are presented in Supplementary Material 2.

#### Training Performance

Overall training sessions, the single leg training capacity totaled 143 patterns, with 127 executed correctly and 16 with errors. For double leg training, a total of 63 patterns were instructed, with 52 executed correctly and 11 with errors. One participant demonstrated an improvement in total trainings capacity from the first to the last session, increasing from four to thirteen patterns for single leg training and from zero to six patterns for double leg training. The performance of the other participants remained stable or declined slightly.

During single leg training, one participant showed an improvement in pattern length from three to five numbers, two participants increased from the first to the second level and one participant increased from 75 to 86 beats per minute. When training with two legs, two participants improved in pattern length from three to four and from zero to five respectively. While only one participant moved up to level two, two participants increased pace by one to six beats per minute. Detailed data on training performance can be found in Table 5.

**Table 5:** Results of Training Performance

ID*	Training Nr.	Single leg						Both legs					
		Capacity			Improvement			Capacity			Improvement		
		Correct patterns	Wrong patterns	Total Patterns	Pattern length	Level**	Bpm***	Correct patterns	Wrong patterns	Total Patterns	Pattern length	Level**	Bpm***
1	1	9	0	9	4	1	75	7	0	7	4	1	75
	2	7	1	8	5	2	75	4	0	4	5	2	70
	3	4	5	9	4	1	75	4	5	9	4	1	75
	4	7	0	7	4	1	86	4	0	4	4	1	76
2****	1	18	0	18	4	1	82	6	0	6	3	1	70
	2	13	0	13	2	2	82	6	0	6	4	1	76
3****	1	7	1	8	3	1	60	0	1	1	3	1	60
4	1	10	1	11	5	2	86	3	0	3	4	1	84
	2	-	-	-	-	-	-	-	-	-	-	-	-
	3	-	-	-	-	-	-	-	-	-	-	-	-
5	1	2	2	4	3	1	80	0	0	0	0	0	0
	2	4	0	4	3	1	80	2	0	2	3	1	80
	3	11	2	13	5	2	80	5	1	6	5	2	80
6****	1	6	0	6	4	1	82	2	0	2	3	1	80
7	1	8	0	8	3	1	70	3	0	3	3	1	70
	2	0	0	0	0	0	0	4	0	4	3	1	60
	3	5	2	7	4	1	60	0	2	2	3	1	60
	4	4	0	4	3	1	65	1	1	2	3	1	60
	5	6	0	6	3	1	70	1	1	2	3	1	60
	6	6	2	8	3	1	60	0	0	0	0	0	0
Total		127	16	143	-	-	-	52	11	63	-	-	-
Mean (SD)		7.06 (±4.17)	0.89 (±1.32)	7.94 (±4.08)	3.78 (±0.81)	-	71.33 (±8.76)	2.89 (±2.27)	0.61 (±1.24)	3.5 (2.48)	3.50 (±0.71)	-	70.89 (±8.76)

**Notes**: *Participants, **Level 1: steps forward, sideways, and backward | Level 2: steps forward, sideways, backward and across the midline | Level 3: steps forward, sideways, backward, across the midline, and crossover, ***Beats per minute, ****Dropouts.

## Discussion

In this study, the feasibility of the motor-cognitive balance training program “StepIt” was examined during acute hospitalization in individuals with TBI. The findings indicate that the study was feasible in terms of recruitment, adherence, and compliance. Adverse events recorded as transient symptom exacerbations (dizziness) during two training sessions were not considered safety risks related to the intervention, as they did not result in negative consequences. According to training recommendations, short-term increases in symptoms like dizziness or nausea are common in individuals with TBI during training and are not expected to adversely affect recovery.^33^ Therefore, these events were not considered indicative of an intervention-related safety concern and the study can be regarded as feasible in terms of safety.

Challenges were observed in participant inclusion and attrition when implementing “StepIt” during acute hospitalization. Of the six predefined feasibility criteria, four were met, while two did not reach the threshold values. Consequently, as shown in Table 1 modifications would be necessary before proceeding to a larger-scale study.

To date, no studies have explicitly examined the feasibility of physiotherapeutic interventions in individuals with TBI during acute inpatient care, although feasibility has been investigated in inpatient and outpatient rehabilitation settings.^34–36^ These studies reported comparable results to the present work, with adherence and compliance rates between 80–100%. The safety findings are also consistent, suggesting that some symptom exacerbation does not pose a health risk. While recruitment rates during acute hospitalization (50–78%) were similar, inclusion rates reported in rehabilitation and outpatient settings (40–64%) were substantially higher than those observed here, which may partly reflect the specific challenges of recruiting and delivering an intervention during acute hospitalization. More specifically, severely affected individuals in the acute phase often do not meet the physical and cognitive demands required for “StepIt,” whereas individuals with mild TBI -although eligible- are frequently discharged within 48–72 hours. Although the anticipated duration of hospitalization can be estimated at the time of screening, it cannot always be reliably predicted, as rapid clinical improvement may lead to earlier-than-expected discharge and consequently premature study termination and incomplete data. Adjusting inclusion criteria based on predicted hospital stay appears impractical: while it could increase inclusion rates, it would likely also raise attrition, leaving the number of complete datasets unchanged. Taken together, the comparison with studies conducted in rehabilitation settings suggests that the main feasibility challenges observed here relate less to adherence or compliance with the intervention itself and more to the organizational constraints of acute hospitalization. Future studies conducted in this setting should therefore consider longer recruitment periods and, where feasible, multicenter recruitment to achieve an adequate sample despite the limited number of eligible patients. Given these challenges, future investigations might consider testing “StepIt” in rehabilitation or outpatient settings, where longer treatment durations and more stable clinical conditions may facilitate recruitment and reduce dropout.

From a cost–benefit perspective, “StepIt” represents a low-cost and adaptable training option for individuals with TBI. Its simplicity and portability enable use in various environments without the need for specialized equipment. However, these advantages do not outweigh the recruitment and screening challenges encountered during acute hospitalization, which substantially increase study effort.

Training performance and user satisfaction results suggest good acceptance of the intervention among individuals with TBI, though this finding should be interpreted cautiously due to the small number of completed sessions and evaluations. Nonetheless, this observation is supported by the high adherence and compliance rates observed, as well as by previous studies reporting beneficial effects of motor-cognitive training in people with TBI.^24–26^,^37^ Aside from the two sessions associated with dizziness, participants reported both cognitive and physical demands during StepIt. The adaptability of the intervention allowed task difficulty to be further increased when needed. A key strength of “StepIt” lies in its flexibility: both cognitive and physical demands can be adjusted individually, and intensity can be tailored through parameters such as pace, task complexity, duration, and frequency, aligning with current recommendations for aerobic and cognitive training.^33^

## Limitations

This study has several limitations. The small sample size and the resulting limited number of complete datasets were primarily due to the single-center design, the relatively short recruitment period of five months, and the inherent constraints of the acute hospital setting. Future studies should consider a multi-center design and a longer recruitment period in inpatient or outpatient rehabilitation environments. Another limitation concerns the collection of user evaluations. For logistical reasons, some questionnaires were distributed with discharge documents rather than immediately after the final training session, which may have introduced recall bias and reduced response rates, necessitating additional effort for data collection. Given these limitations, the results should be interpreted with caution and not generalized beyond the study context. Nevertheless, this study provides valuable insights for both research and clinical practice. For research, it establishes a foundation for a larger trial evaluating the effectiveness of “StepIt”. A randomized controlled trial comparing “StepIt” with standard care in a larger sample of patients with TBI (preferably in a rehabilitation or outpatient setting) is warranted to determine efficacy. Conducting such a study as a multi-center trial could enhance generalizability and facilitate recruitment. For clinicians, “StepIt” represents a practical and resource-efficient option for addressing motor and cognitive impairments after TBI, as it can be implemented flexibly and without costly equipment.

## Conclusion

The present findings indicate that StepIt is feasible in terms of recruitment, adherence, compliance, and safety during acute hospitalization in people with TBI, while challenges were identified regarding inclusion and attrition. Given the small sample size, these findings should be interpreted cautiously and cannot be generalized beyond the present study context. Future studies in the acute-care setting should consider methodological adaptations to address recruitment challenges, including longer recruitment periods and multicenter recruitment. Further research in larger samples is required to evaluate the effectiveness of StepIt.

## Data Availability

All datasets generated for this study are included in the article. Further inquiries can be directed to the corresponding authors.
